# The Development of Intellect in Emerging Adults: Predictors of Longitudinal Trajectories

**DOI:** 10.3390/jintelligence12110113

**Published:** 2024-11-08

**Authors:** Patrick Mussel

**Affiliations:** 1 Division for Personality Psychology and Psychological Assessment, Department of Education and Psychology, Freie Universität Berlin, Habelschwerdter Allee 45, 14195 Berlin, Germany; patrick.mussel@fu-berlin.de; 2 Division for Psychological Diagnostics and Differential Psychology, Psychologische Hochschule Berlin, Am Köllnischen Park 2, 10179 Berlin, Germany

**Keywords:** intellect, epistemic behavior, cognitive ability, social investment principle

## Abstract

Intellect is an important personality trait, especially with regard to the prediction and explanation of intellectual performance, such as occupational or academic success. However, much less is known about the development of Intellect. I present results from a longitudinal study spanning eight years to investigate changes in Intellect during a critical period: the transition from school to vocation. The study is based on a large and heterogeneous sample with up to 1964 participants. Using a facet approach, I investigate predictors of longitudinal trajectories theoretically derived from construct definition, including subjective and objective attributes of education and profession; attitudes regarding the malleability of personality traits; as well as personality traits beyond Intellect, especially intelligence. Results reveal some support for the social investment principle according to neo-socioanalytic theory, as epistemic job demands and epistemic leisure activities predicted the increase in Intellect over time. The study contributes to our understanding of the development of personality traits related to intellectual achievement, including important internal and external predictors of longitudinal trajectories.

## 1. Introduction

Intellect, an aspect of the domain Openness/Intellect of the Big Five model, has important implications for various life outcomes, such as academic performance (Bertrams and Dickhäuser 2009; Strobel et al. 2019; von Stumm et al. 2011), job performance (Mussel 2013b), entrepreneurship (Heinemann et al. 2022; Lievens et al. 2021), creativity (Harrison and Rouse 2014; Mussel et al. 2015), and health outcomes (Grass and Strobel 2024; Strobel et al. 2021). Levels of Intellect have also been proposed to affect the development of cognitive abilities (Ackerman and Heggestad 1997; Ziegler et al. 2015; Ziegler et al. 2012). However, much less is known about how Intellect develops itself. The present study reports results from a longitudinal study spanning eight years in which trajectories of Intellect have been investigated during a critical time period: the transition from school to work. The focus on a single narrow trait according to a facet perspective allowed for deducing theoretical hypotheses from the construct definition regarding potential predictors of personality development.

### Background

The neo-socioanalytic theory (Roberts and Wood 2006) conceptualizes personality change as a bottom-up process (Roberts and Mroczek 2008). The theory posits that personality may change in reaction to the environment as individuals take on and invest in new social roles (termed the social investment principle). Recurrent changes in behavioral patterns according to new social roles are believed to reflect changes in personality over time (Baumert et al. 2017; Geukes et al. 2018; Quintus et al. 2021; Roberts 2018; Wrzus and Roberts 2017). In addition, individuals select themselves into environments that match their personality, which has been termed as the corresponsive principle (Caspi and Roberts 2001; Jackson et al. 2012; Noftle and Fleeson 2010; Roberts and Wood 2006; Wrzus et al. 2016). Thus, changes in Intellect may be the result of investment in such new social roles that require behavior that reflects the construct’s definition. 

Intellect is a dispositional individual difference variable involving behavior, intentions, affect, attitudes, and mental processes related to intellectual performance. People high in Intellect can be described as smart, clever, and curious; they enjoy thinking, problem-solving, learning, and cognitively demanding tasks (DeYoung et al. 2012; Goldberg 1992; Mussel 2013a). According to the Big Five personality structure, Intellect can be classified as a subdimension (or facet) of the personality factor of Openness/Intellect (DeYoung et al. 2007). The compound label Openness/Intellect constitutes an empirically derived dimension that displays individual differences and describes processes that reflect cognitive and perceptual exploration (see DeYoung 2015 for a review). Across the life span, Intellect shows inverted U-shaped trajectories, with increases in younger adults and decreases in older adults (Bruinsma and Crutzen 2018; Roberts et al. 2006), while the meaning of the construct may be the same across the life span (Soubelet and Salthouse 2017). The internal structure of Intellect can be described according to the Theoretical Intellect Framework, which proposes two dimensions: Process, which relates to either Seeking epistemic situations or Conquering them once they are encountered; and Operation, relating to the content of the epistemic behavior, thereby delimitating the operations Think, Learn, and Create (Mussel 2013a). Intellect can be considered the personality counterpart of cognitive abilities; whereas cognitive abilities refer to ability, which is the “can do” aspect of performance, Intellect refers to the personality trait and motivational component, namely, the “will do” or “typically do” aspect (Cronbach 1960). 

Combining theoretical assumptions about the bottom-up process of personality development as outlined by the neo-socioanalytic theory and definitional attributes of the trait Intellect, I assumed that the development of Intellect may be shaped by features of the environment that foster epistemic behavior, such as thinking, learning, creating, or problem-solving. Examples include attending a training course during leisure time, learning a complex theory at university, or being responsible for a complex project at work. 

Some preliminary evidence supports this hypothesis. Woods et al. (2020) report that an investigative work environment fosters the development of Openness/Intellect. Golle et al. (2019) compared individuals who accomplished academic compared to vocational training and found that the former led to a stronger increase in investigative interest and a decrease in conscientiousness. Askim et al. (2022) found that six out of seventeen work characteristics predicted personality change in the Big Five; for example, openness increased when workers experienced job control, such as the presence of freedom of choice between alternatives; as well as when perceiving empowering leadership, such as receiving encouragement from the leader to express their opinions and to develop themselves. Zheng et al. (2024) found that job characteristics derived from job titles predicted personality change, especially for openness. In addition, leisure activities have been found to be associated with personality change, such as changes in extraversion being predicted by socializing during leisure time, even though no effects were found for openness (Sander et al. 2021).

In the present study, I investigate the hypothesis of an influence of epistemic environmental characteristics on the development of Intellect more directly by measuring four indicators of work and leisure attributes: First, job-related tasks regarding literacy and numeracy according to the international codebook PIAAC (Rammstedt et al. 2013). Second, job demands, according to trait activation theory, using a measure that obtains job characteristics on the task, social, and organizational levels that have the potential to activate Intellect (Mussel and Spengler 2015). Third, the area of educational or vocational activity using the RIASEC taxonomy (Holland 1997), thereby comparing activity in the investigative area with activity in other areas (e.g., social, conventional). Fourth, epistemic leisure activities, such as reading books or attending voluntary training. Thus, the first hypothesis of the present study can be outlined as follows:

**Hypothesis** **1.** *High compared to low levels in epistemic environmental characteristics predict a stronger increase in Intellect over time.*

In addition to environmental characteristics, I also investigated an internal predictor of changes in Intellect in terms of core beliefs (Mussel 2024). Core beliefs are generalized cognitions that people hold that reflect important experiences that they made while pursuing need-fulfilling goals (Dweck 2018), and such beliefs have been found to predict personality trait change (Mussel et al. 2023). Here, I investigate beliefs that have been referred to as mindset (Dweck 2012), which describes cognitions that individuals hold concerning the malleability of personality traits. In particular, relating to the general debate about nature and nurture, Dweck distinguished between a fixed mindset, which is characterized by the belief that personality traits and intelligence are genetically determined, versus a growth mindset, which describes individual beliefs that personality and intelligence are malleable due to experiences or effort. From this definition, one might conclude that a growth mindset also predicts an actual change in personality traits, as individuals might voluntarily seek opportunities to change their personality. Indeed, Park et al. (2020) found that a growth mindset predicts the development of grit. On the contrary, Hudson et al. (2021) found no effect of mindset on personality trait change, yet they only investigated a four-month period of time. Given the few studies on the topic and the mixed results, I investigate whether mindset might predict the personality trait Intellect across a longer period of time, leading to the second hypothesis:

**Hypothesis** **2.** *Growth, compared to a fixed mindset, predicts a stronger increase in Intellect across time.*

As a third objective, cognitive abilities were investigated as predictors of change in Intellect. As outlined in the environmental success hypothesis in the OFCI model (Ziegler et al. 2012; Ziegler et al. 2018), individuals with high compared to low cognitive ability are more likely to succeed in cognitively challenging situations, which results in more positive evaluations of cognitive effort and might, over time, affect levels of Openness/Intellect (e.g., enjoyment of thinking and problem-solving). In line with this principle, Bergold and Steinmayr (2024) found that cognitive ability predicts change in investment traits. In a second study, there was partial support for a predictive effect of cognitive ability on changes in hope for success but not in other investment traits (Bergold et al. 2023). Accordingly, the third hypothesis posits:

**Hypothesis** **3.** *High compared to low levels in cognitive ability predict a stronger increase in Intellect across time.*

Finally, this study also investigates the reverse effect, i.e., the personality trait Intellect as a predictor of change in epistemic environmental characteristics, mindset, and cognitive ability. These analyses are based on the correspondence principle (Roberts 2018) according to which individuals select themselves into environments that match their personality (Jackson et al. 2012; Wrzus et al. 2016). The effect of change in cognitive ability due to levels in Intellect corresponds to the environmental enrichment hypothesis (Ziegler et al. 2012), which has previously received some support (Raine et al. 2002; Trapp and Ziegler 2019; Ziegler et al. 2015), even though not consistently (Mussel 2022). The investigation of a potential effect of levels of Intellect on trajectories in the aforementioned variables can be summarized as a research question: 

Research Question 1: Do levels of Intellect predict the development of epistemic environmental characteristics, mindset, and cognitive ability?

This study reports longitudinal results for a large sample of adolescents during a critical period, the transition from school to vocational training or tertiary education and further to their first job. This period is generally characterized by large transitions, which have been found to foster personality change and thus provide an opportunity to further understand the drivers underlying personality change. 

## 2. Materials and Methods

### 2.1. Panel and Procedure

Data were collected from participants of the German Personality Panel (GePP, Mussel 2021). The panel was established by contacting individuals who formerly (in 2016 or 2017) participated in an online, free-of-charge career counseling test (berufsprofiling.de). In 2018, participants who agreed to be contacted again were asked whether they would like to participate in a scientific panel study. Since then, participants have been contacted approximately once a year and asked to take a personality test, taking between thirty and forty-five minutes. Participants received feedback on their results and were compensated via vouchers with 5 to 10 Euros. The test batteries included a variety of test formats, including self-report personality tests, cognitive tests, and social-cognitive tests, as well as questions regarding live events and their personal situation; see (Mussel 2021) for details. For the present study, I use data from all measurement occasions on which a measure for Intellect was available, particularly from the career counseling test in 2016 and 2017 (from here on referred to as T1) and the panel study measurements in 09/2018 (T2); 10/2019 (T3); 11/2020 (T4); 11/2021 (T5); and 06/2024 (T6). Thus, the data span a time interval of more than 8 years. 

### 2.2. Sample

A total of 11,816 individuals participated in the career counseling in 2016/2017. According to the scope of the counseling, most individuals presumably had prepared their first career decision when taking the career counseling. When contacted in the course of the panel study, *N* = 1964 agreed to participate in the study and took at least one test. Regarding the six measurement time points considered in this study, *N* = 169 took part at all six measurement time points; *N* = 270 at five; *N* = 318 at four; *N* = 381 at three, and *N* = 791 at two measurement time points. At the time of T1, most panel members were between 14 and 22 years old (mean age: *M* = 17.3; *SD* = 2.3), 65% were female, and 35% were male. According to a subsample for whom the year of graduating from school was assessed (*N* = 419, obtained at T3), 74% were still at school when taking the career counseling. The test was applied in the German language.

### 2.3. Measures

A full description of all measures obtained at all measurement occasions is available online (Mussel 2021). Data from the following measures are reported in the present study:

The 10-item Work-Related Curiosity Scale (WORCS; Mussel et al. 2012): Responses are given on a 7-point Likert scale ranging from “does not apply at all” (1) to “partly” (4) to “fully applies” (7). An example item is “I am eager to learn”. The test was applied on all measurement occasions. On average, across the six measurement time points, the reliability coefficients were α = 0.86 and ω = 0.89. Re-test reliability between measurement time points was, on average, *r*_tt_ = 0.60.

The 24-item Intellect Scale (Mussel 2013a): Responses are given on a 7-point Likert scale, as above. An example item is “I enjoy solving complex problems”. The test was applied on measurement occasions T2, T3, T4, T5, and T6. On average, across the five measurement time points, the reliability coefficients were α = 0.94 and ω = 0.95. Re-test reliability between measurement time points was, on average, *r*_tt_ = 0.65.

Both the Intellect Scale and the WORCS are indicators of Intellect (Mussel 2010, 2013a). In the present study, the two scales correlated, on average, with *r* = 0.86 and were thus aggregated. Therefore, the mean of all items of the Intellect Scale was scaled to the mean of the WORCS at T2 (the first measurement occasion on which both measures were applied). 

For the assessment of environmental enrichment, I used four indicators of epistemic behavior at work and during leisure time. First, epistemic behavior at work was assessed using 20 items from the PIAAC international codebook (Rammstedt et al. 2013). These items assess skill use on the job regarding literacy and numeracy. An example item is “reading instructions and manuals”. Responses are given on a 5-point Likert scale with the response options “never” (1), “less than once a month” (2), “at least once a month, but not once a week” (3), “at least once a week, but not daily” (4), and “daily” (5). Epistemic behavior at work (PIAAC) was assessed on measurement occasions T2, T5, and T6. In the present study, the reliability coefficient was, on average, α = 0.84 and ω = 0.88, and the retest reliability was, on average, *r*_tt_ = 0.34 (note that low correlations may also reflect changes in work and leisure behavior across time, for example when graduating from high school or university or when work activities at work vary, indicating that it might constitute a situational rather than a stable variable). 

Second, work characteristics with regard to epistemic behavior were assessed using eight items from the instrument assessing curiosity-activating work characteristics (Mussel and Spengler 2015). The instrument assesses epistemic job demands on the task, social, and organizational levels according to trait activation theory (TAT; see Tett and Burnett 2003). An example item is “My current education/occupation requires creative solutions for new problems”. Responses are given on a 7-point Likert scale ranging from “does not apply at all” (1) to “partly” (4) to “fully applies” (7). Epistemic job demands (TAT) were assessed on measurement occasions T2, T3, T4, T5, and T6. In the present study, the reliability coefficient was, on average, α = 0.81 and ω = 0.87, and the retest reliability was, on average, *r*_tt_ = 0.37.

Third, the type of current educational program/occupation was assessed via a single-item self-report item according to the RIASEC model (Holland 1997). Participants were given descriptions for the six vocational categories, e.g., “Practical-technical (e.g., craftsmen, machine operators, farmers and foresters, animal keepers, technical draughts men)”. Answers for investigative were coded as 1, and all others were coded as 0. Investigative education/occupation (RIASEC) was assessed on measurement occasions T3, T4, T5, and T6.

Fourth, epistemic leisure activities were assessed with four ad hoc-developed items (e.g., reading books, attending advanced training, and acquiring new knowledge). Responses were given on a 5-point Likert scale with the response options “never” (1), “seldom” (2), “sometimes” (3), “often” (4), and “very often” (5). Epistemic leisure activities were assessed on measurement occasions T2, T3, T4, T5, and T6. In the present study, the reliability coefficient was, on average, α = 0.63 and ω = 0.71, and the retest reliability was, on average, r_tt_ = 0.46.

Mindset was assessed via three items (Dweck 2012). Responses are given on a 7-point Likert scale, as above. An example item is “Personality traits of a person are something that can hardly be changed”. All three items were reverse scored according to x′ = 8 − x to reflect a growth (compared to a fixed) mindset. The test was applied on measurement occasions T2, T3, T4, and T6. On average, across the four measurement time points, the reliability coefficients were α = 0.80 and ω = 0.81. Re-test reliability between measurement time points was, on average, *r*_tt_ = 0.40.

Cognitive ability was assessed with three short indicators assessing numeric, figural, and verbal abilities (HR Diagnostics n.d.). Tests were assessed at T1, T2, T3, and T5. For numerical abilities, a 10-item number series test was used. Each item consists of a series of numbers that must be completed. Answers are given as free text, and the correct answer is scored as “1”. In the present study, the reliability coefficient was, on average, α = 0.82 and ω = 0.84. The average retest reliability was *r*_tt_ = 0.66.

For figural abilities, a 20-item matrix test was used. Each item consists of a classical 3 × 3 matrix, with the last element substituted by a question mark. Participants must choose an object from a list of six objects that logically fits, such that the underlying role is preserved. Correct answers are scored as “1”. In the present study, the average reliability coefficient was α = 0.77 and ω = 0.79. The average retest reliability was *r*_tt_ = 0.64. For verbal abilities, a 28-item measure to assess vocabulary was used. Each item consists of five words, four of which do not exist. Here, participants must choose the word that exists. Correct answers are scored as “1”. In the present study, the average reliability coefficient was α = 0.86 and ω = 0.87. The average retest reliability was *r*_tt_ = 0.72.

Indicators for all three subtests were standardized according to mean and standard deviation at T1 and subsequently aggregated. For the aggregated score, the reliability coefficient across the three subtests was α = 0.73 and ω = 0.74, and the retest reliability was, on average, *r*_tt_ = 0.73.

For the assessment of careless responding, a single self-reported item asking participants whether they had worked on the test carefully (with two options: yes and no) was obtained. Participants were instructed that their answer would not affect obtaining the financial incentive. The careless response indicator was obtained at T2, T3, T4, T5, and T6.

### 2.4. Careless Responding, Missingness, and Attrition

At T1, six individuals had no data on any of the above-mentioned variables (except demographics) and were subsequently dropped. The number of individuals who answered with “no” on the careless responding item were: *N* = 41 (2.4%) at T2; *N* = 27 (2.5%) at T3; *N* = 5 (0.8%) at T4; *N* = 4 (0.9%) at T5; and *N* = 2 (0.3%) at T6. These cases were subsequently excluded from further analysis. 

For the statistical analyses, missing data were excluded listwise per measurement time point and per analyses. As not all measures were applied on all measurement time points and not all individuals participated in all measurement time points, this resulted in the following final sample size, depending on the analysis: *N* = 1930 for change in Intellect according to demographic variables; *N* = 1447 for epistemic behavior at work (PIAAC); *N* = 1646 for epistemic work characteristics (TAT); *N* = 1213 for type of current occupational program/occupation (RIASEC); *N* = 1651 for leisure activities; *N* = 1790 for mindset; and *N* = 1930 for cognitive ability. 

### 2.5. Statistical Analyses

All statistical analyses were carried out in R 4.4.1 and RStudio 2024.4.2 using the packages psych (Revelle 2022) and lme4 (Bates et al. 2014). I analyzed the data using linear mixed effects models. These models are generally equivalent to latent change score models (Newsom 2017), yet are more flexible for analyzing data with varying intervals between measurement time points, missing measurements, and individually varying time points (which varied up to 2 years between T1 and T2). Additionally, latent change score models with phantom variables are problematic when missing measurement time points pertain to the first occasion, T1, which was the case for 2 out of 4 variables in the present study. 

Time-relevant variables were centered, and all other variables were standardized prior to the analyses such that a fixed effect indicated standardized change per year. Intellect served as the dependent variable. Fixed effects were estimated for age at T1; gender; time difference between measurement occasions; and the predictor variable. Additionally, random slopes for time and random intercepts were estimated. Age at T1 and gender were included as level 2 control variables to account for age differences at the start of the study and gender differences in Intellect. The time difference between the respective measurement occasions and T1 served as a level 1 fixed effect to investigate changes in Intellect across time. As T1 occurred within a time range of two years, time difference was included as a continuous predictor. Six separate models were estimated, one for each of the predictor variables (epistemic behavior at work (PIAAC); epistemic work characteristics (TAT); type of current occupational program/occupation (RIASEC); leisure activities; mindset; and cognitive ability, respectively). Levels of the predictor variables were included as level 2 variables according to aggregated scores across all available measurement time points. 

As an additional analysis, the reverse effect of Intellect on change in the six (formerly) predictor variables was estimated. Here, Intellect served as a level 2 predictor variable and each of the six other variables as dependent variables in separate analyses. All other aspects of the model were equivalent, as described above. 

### 2.6. Preregistration and Open Science Statement

I report how I determined the sample size, all data exclusions, all manipulations, and all measures in the study (Simmons et al. 2012). The present study was not formally preregistered. When planned in 2016, pre-registration was not yet common. However, all hypotheses and methods were outlined in a third-party funding proposal, which was submitted in 2016 and is available via OSF (https://osf.io/zeq5b//; accessed on 2 September 2024). All data and scripts are available online via https://osf.io/jw3t9/ (accessed on 5 October 2024). Sources for materials for all measures assessed can be found at https://doi.org/10.17605/OSF.IO/7W9YJ (accessed on 5 October 2024). 

## 3. Results

Descriptive results and bivariate associations can be found in Table 1. As noted above, curiosity and Intellect were highly associated and show a similar pattern with other variables. Negative correlations with age at T1 indicate that male participants had, on average, higher scores on curiosity and Intellect compared to female participants. The four indicators of epistemic behavior at work were positively associated with curiosity and Intellect (on average *r* = 0.26), as were epistemic leisure activities (on average *r* = 0.43). Associations with cognitive ability were also positive yet differed between the two scales, with stronger correlations for curiosity compared to Intellect. A closer inspection of the data revealed that the stronger correlation between curiosity and cognitive ability was only found at T1 but not at other measurement time points. Thus, the difference between the two scales can be explained by the fact that the Intellect scale was not applied at T1 (correlations were similar at other measurement time points). 

**Figure 1:**
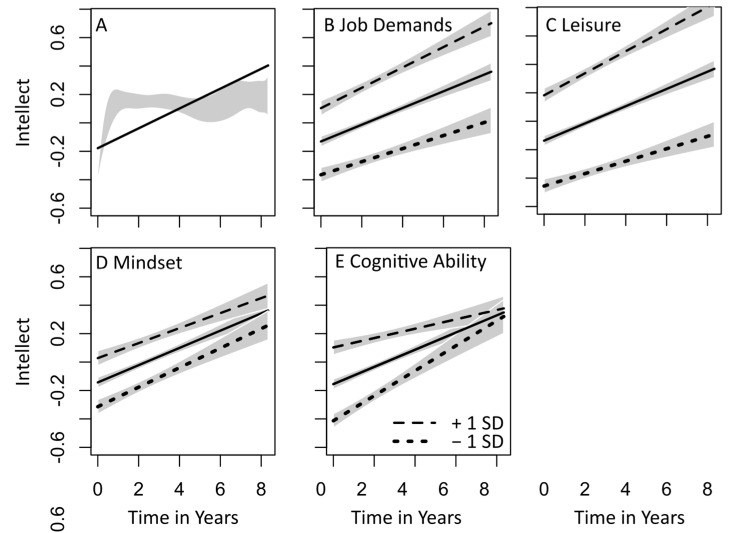
(**A**) Predicted linear trajectories in standardized Intellect. (**B**–**E**) Effects of significant predictors (dashed for 1 standard deviation above the mean and points for 1 standard deviation below the mean).

Results for the fixed-effect estimates of the mixed model analyses are shown in Table 2. The upper part shows all effects for the base model, predicting Intellect according to age at T1, gender, and time. Intellect was not related to age at T1. However, the fixed effect for time was significant. The positive estimate of 0.09 indicates that Intellect increased across time by .09 standard deviations per year. The predicted levels of Intellect as a function of time are depicted in Figure 1A. Visual inspection of the means indicated that the increase was strongest within the first two years (i.e., between T1 and T2). Levels of Intellect did, on average, not change between two and five years. From five years onwards, levels increased again, even though less strongly. This pattern is illustrated as a grey-shaded area in Figure 1A, reflecting the 95% confidence intervals of the polynomial fit (for n = 7). 

The fixed effect for gender was also significant, reflecting higher scores for male compared to female participants (see also descriptive results in Table 1). Additionally, the negative and significant interaction between time and gender indicated that the difference increased over time. Finally, the interaction between age at T1 and time was significant. The negative effect estimate indicates that increases in Intellect across time were larger when participants were younger at T1, reflecting the pattern of stronger increases in Intellect in the younger age of adolescents. According to these results, age at T1 and gender were included in all following analyses to control for the effect of demographic variables. 

Next, the mixed model described above was repeated by additionally adding the six predictors one at a time in separate analyses. In line with the bivariate results reported in Table 1, all main effects were significant on *p* ≤ .001. Results for the influence of the predictors on the development of Intellect (i.e., the interaction between a predictor and time) are depicted in the lower part of Table 2. I found no effect on the development of Intellect for epistemic behavior at work, as assessed via the PIAAC items, as well as for individuals who indicated that their education or occupation was in the investigative area, compared to other areas (RIASEC). However, epistemic job demands, assessed according to Trait Activation Theory (TAT), significantly predicted trajectories in Intellect (see also Figure 1B). The positive fixed-effect estimate indicates that the effect was in the expected direction, i.e., stronger increases in Intellect for higher compared to lower levels in epistemic job demands. 

The interaction between epistemic leisure activities and time on Intellect was also significant and in the expected direction, indicating that higher compared to lower levels in epistemic leisure activities predicted a stronger increase in Intellect (see Figure 1C). The interaction between time and mindset was also significant, albeit in the opposite direction than expected, i.e., a stronger increase was found for individuals with a fixed (compared to growth) mindset. 

Surprisingly, a significant and negative fixed-effect estimate was found for the interaction between cognitive ability and time; see Figure 1E. Contrary to the positive bivariate correlation between Intellect and cognitive ability, the increase in Intellect was stronger for low compared to high levels of cognitive ability. A closer inspection of the results indicated that this pattern was due to a stronger correlation between cognitive ability and Intellect at T1 (*r* = 0.26) compared to all other measurement time points (all 0.14 < *r* < 0.18).

Finally, given that all predictors were assessed at multiple measurement time points, I also report the reverse effect of levels of Intellect on change in the six (formerly) predictor variables. As above, the models include age at T1, gender, and time as fixed effects, in addition to Intellect. Results for the interaction term between Intellect and time are depicted in Table 3, each referring to a separate mixed model analysis. Two significant effects were found: First, levels of Intellect predicted trajectories in leisure activities, with a stronger increase in epistemic leisure activities for high compared to low levels of Intellect (marginally significant at *p* = .054). Second, the interaction term between Intellect and time was significant for cognitive ability as the dependent variable, indicating that levels of cognitive ability increased more strongly for individuals with high compared to low Intellect. 

## 4. Discussion

Personality traits are characterized by both stability and change during the life span (Roberts et al. 2001). The neo-sociogenomic model of personality traits posits that stability in personality traits is due to genetic factors, whereas change arises due to changes in the environment, mediated by pressure on personality states (Roberts 2018). These states, when occurring repeatedly, become internalized, automatized, and generalized and might alter the genetic influence due to epigenetic processes. The present study investigated hypotheses derived from the neo-sociogenomic model by assessing characteristics of the environment to predict individual trajectories in personality. Employing a facet perspective and thus focusing on a narrow trait allowed for the deduction of hypotheses about environmental characteristics from the definition of the trait. 

The results partially supported the hypotheses. From the four epistemic environmental characteristics that were assumed to predict trajectories in Intellect, two showed significant and expected relations with change in trait Intellect: First, educational or occupational epistemic job demands, such as tasks requiring learning new skills, coming up with creative solutions, or adjusting to a new situation, or colleagues who discuss a lot, teach one new skills, and make one think and learn. Second, epistemic leisure activities, such as reading books, voluntarily attending further training courses, or learning new things. Higher levels in these epistemic environmental characteristics predicted a stronger increase in Intellect over time, thus supporting assumptions from neo-socioanalytic theory. 

On the other hand, no support was found for two other indicators: skill use on the job regarding literacy and numeracy according to PIAAC and type of current educational program/occupation according to the RIASEC model. As a potential methodological reason for the lack of effect of the former, some of the items assessing literacy and numeracy might not have discriminated well between high and low epistemic environments, especially for our sample. For example, while reading and writing emails, reading books or instructions, or using simple algebra might well distinguish jobs characterized by mental versus manual work, these skills will be relevant for almost all jobs during training and apprenticeship. Regarding the RIASEC indicator, the categorization into two dichotomic groups (epistemic vs. non-epistemic) according to investigative and non-investigative areas might have been too rough, missing nuanced details between educational programs and jobs. Additionally, there are many complex jobs that are not characterized as investigative, such as entrepreneurs (from the domain enterprising), statisticians (from the domain conventional), or clinical psychologists (from the domain social). Given that estimates for both indicators were in the expected direction, the weak and non-significant prediction might have been due to these methodological reasons. 

Additionally, the study investigated a potential reverse effect from trait Intellect on changes in epistemic characteristics. Such an effect would be in line with the correspondence principle (Roberts 2018), according to which individuals select themselves into environments that match their personalities. I only found one marginally significant effect for leisure activities, indicating that higher levels in trait Intellect might tendentially predict stronger pursuit of epistemic leisure activities, such as reading books. The lack of effects for the education and work-related environmental characteristics might reflect that occupational choice depends on a variety of factors in addition to precipitating correspondence with levels of trait Intellect, such as starting an educational program because it is available in a certain region or because it allows for making money. 

Overall, these results show some support for the assumed processes of selection and socialization of the environment in relation to trait change. The strong bivariate correlations between Intellect and the epistemic environmental variables investigated in this study suggest that additional processes might take place, such as genetic effects or non-investigated third variables that affect both trait Intellect and epistemic environmental characteristics.

The hypothesis for mindset was not supported. Indeed, the results showed an opposite effect compared to what was expected, with a stronger increase in trait Intellect for individuals with a fixed (compared to a growth) mindset. The construct of mindset has been developed against the background of the nature–nurture debate (Dweck 2012). It assesses the beliefs of individuals concerning the extent to which personality traits are predisposed due to genetic factors versus malleable due to experiences that individuals make or effort that they invest. A growth mindset has been shown to be beneficial for a variety of life outcomes, such as mental health (Tao et al. 2022) or academic achievement (McCutchen et al. 2016). The present hypothesis was derived from construct definition, assuming that a belief that personality may be changed might result in actual change, e.g., due to self-initiated personality change, willingness to change or adapt, or voluntarily seeking new experiences. However, Hudson et al. (2021) already found disconfirming evidence and noted that “your personality does not care whether you believe it can change” (p. 340). As an alternative hypothesis for the influence of mindset on personality change, future research may benefit from taking a differentiated view on the personality traits that are expected to be changed. As such, there may be positive effects for some and negative effects for other traits. For example, taking the prevailing evidence regarding a positive relationship between growth mindset and academic achievement and the effect on the development of grit (Park et al. 2020), one may assume that a growth mindset results in higher states of willingness to achieve, which might influence traits such as conscientiousness (rather than Intellect/Openness). Additionally, it should also be mentioned that mindset showed acceptable internal consistency, yet only low retest reliability. Low stability might indicate that mindset is rather a situational variable, thus making it less suitable as a level 2 predictor for personality change. 

Regarding cognitive ability, results disconfirmed the assumed positive influence of cognitive ability on change in Intellect. Closer inspection of the data revealed that this effect was fully driven by stronger correlations between Intellect and cognitive ability on the first time point compared to all further measurement time points. Given this pattern and prior evidence in favor of the original hypotheses (e.g., Bergold and Steinmayr 2024), the present results should not lead to a premature disproof of the hypotheses. A possible explanation for the current findings may lie in the personal situation in which the participants were at different measurement time points: On the first measurement time point, while most participants were still at school, the self-concept of Intellect might have been more strongly grounded in scholastic achievement, which itself is strongly related to cognitive abilities. After finishing school, individuals pursued diverse paths, from taking a year off for traveling to working or studying. Thus, the self-concept of behavioral, emotional, motivational, and cognitive tendencies related to Intellect, such as enjoyment of thinking, learning, and creating, might now be less strongly related to academic performance and, thus, to cognitive performance. As another possible explanation, ceiling effects could have occurred such that individuals with high cognitive ability have largely exhausted their potential, whereas those with lower intelligence still have room for motivational improvement.

Interestingly, I found a reverse effect of Intellect on changes in cognitive abilities, which is in line with the environmental enrichment hypothesis of the OFCI model (Ziegler et al. 2012). Thus, levels of cognitive ability increased more strongly for individuals with high Intellect compared to those with low Intellect. Future studies are needed to better understand the processes underlying this effect, especially by taking interests and situational states into account (Ziegler et al. 2018). 

Changes in personality traits often occur during so-called critical phases, such as engaging in a new relationship, starting an apprenticeship, changing jobs, or moving to a different location (Bleidorn 2012; Hutteman et al. 2015; Jackson et al. 2012; Wrzus and Neyer 2016). Accordingly, lower rank order consistencies in personality traits have been reported during adolescence and young adulthood, a period that is characterized by marked changes in life situations (Roberts et al. 2001; Roberts and DelVecchio 2000). Results from the present study support this notion, as well as prior empirical evidence on changes in level of Intellect (Bruinsma and Crutzen 2018), reflected in substantial changes in Intellect during a period of eight years. Additionally, during this period, the strongest change in Intellect was found at the beginning, followed by a smaller change at the end of the period, whereas the middle part was characterized by a plateau. According to the recruitment strategy of the German Personality Panel, most participants were presumably in their last year of school when taking the career counseling test (the first measurement occasion of the panel study) while preparing for their first vocational choice. Thus, the change between the first and the second measurement points reflects changes in personality traits during the transition from school to vocational training or studying. On the other hand, changes at the end of the period, between four and eight years after the first measurement point, might reflect the transition from training and studying to their first job. One reason for the more decent increase during this phase might be the different length of qualification: while vocational training usually takes two to three years, a bachelor’s degree takes three years and a master’s degree five years, in addition to individually varying duration. Thus, the pattern of results is in line with the idea of changes during critical phases, underscoring the idea of the social investment principle, according to which changes in social roles foster personality development (Roberts and Wood 2006).

In addition to what has been already mentioned above, the following three limitations shall be stated: First, due to restrictions in testing time and the design of the study (recruitment of participants according to a career counseling test), not all variables could be measured on all measurement time points. This also restricted the use of more common statistical methods for analyzing the longitudinal data, such as cross-lagged panel models, latent change score models, or latent growth models (even though results for mixed models are generally equivalent). Given the openly available data, readers are invited to pursue different analysis strategies to analyze the data. Particularly noteworthy is that on the first measurement time point, only one out of two indicators of Intellect were available; however, given average manifest correlations of *r* = 0.86 between the two indicators, they can be assumed to measure the same construct. Second, also relating to the design of the study, the testing situation was different on the first compared to all other measurement time points, which might have affected both levels as well as correlations between measures. However, all measurements occurred under voluntary, low-stakes testing conditions. 

A third limitation is potential attrition effects. The data for the first measurement occasion were gathered through a non-profit self-assessment test intended to help individuals explore post-graduation educational and occupational opportunities. Therefore, the sample might be prone to selection effects and confounding preexisting differences: only emerging adults who were concerned about their future might have taken the test in the first place, potentially leading to variance restriction. In addition, those participants who took the self-assessment voluntarily decided to participate in a research study (or not), which might have contributed to the higher rate of female compared to male participants in the research study compared to the self-assessment. Given the longitudinal design, further selection effects might have occurred, such as a higher rate of participation for individuals with high compared to low conscientiousness scores, leading to further variance restriction in some variables. 

## 5. Conclusions

The present study provides evidence on the developmental trajectories in Intellect in a large-sample longitudinal study spanning 8 years during a critical time period, the transition from school to work. Hypotheses for predictors on Intellect trajectories were deduced from contemporary theories on personality development in combination with definitions of the trait. Some support was found for the effect of epistemic environmental characteristics on growth in Intellect, thereby supporting the neo-sociogenomic model of personality development. Additionally, support was found for the environmental enrichment hypotheses, whereas other hypotheses, especially those relating to mindset, were not supported.

## Figures and Tables

**Table 1:** Descriptive results and bivariate correlations between all study variables. Gender is coded as 0 = male and 1 = female. Data on the personality measures were aggregated across measurement points, if available. Correlations significant on *p* < .05 are depicted in italics.

	n	M	SD	1	2	3	4	5	6	7	8	9
1. Age at T1	1961	17.30	2.3									
2. Gender	1962	0.65	0.5	−0.03								
3. Curiosity (WORCS)	1933	4.75	0.8	*0.05*	*−0.16*							
4. Intellect (Intellect Scale)	1793	4.84	0.8	0.01	*−0.16*	*0.86*						
5. Epistemic behavior at Work (PIAAC)	1448	2.72	0.6	*−0.15*	*−0.14*	*0.25*	*0.25*					
6. Epistemic job demands (TAT)	1649	4.63	0.9	*−0.09*	−0.03	*0.30*	*0.30*	*0.40*				
7. Investigative education/occupation (RIASEC)	1215	0.27	0.4	*−0.10*	−0.01	*0.26*	*0.23*	*0.15*	*0.18*			
8. Epistemic leisure activities	1654	2.95	0.7	−0.04	*−0.06*	*0.42*	*0.43*	*0.32*	*0.29*	*0.18*		
9. Mindset	1793	3.80	1.3	−0.02	*−0.05*	*0.20*	*0.13*	−0.01	*0.10*	*0.14*	*0.22*	
10. Cognitive ability	1964	−0.03	0.9	−0.03	−0.03	*0.31*	*0.16*	0.04	*0.14*	*0.30*	*0.13*	*0.20*

**Table 2:** Results from mixed model analyses for predicting Intellect by the variables depicted in the first column. Results in the lower half are from separate analyses.

Fixed Effect	Estimate	*F*	*p*
Age at T1	0.019	1.4	.23
Time	0.090	264.4	.00
Gender	−0.302	56.1	.00
Age at T1 : Time	−0.004	11.2	.00
Age at T1 : Gender	−0.017	1.0	.33
Time : Gender	−0.020	4.2	.04
Age at T1 : Time : Gender	−0.005	1.7	.20
Time : Epistemic behavior at work (PIAAC)	−0.002	0.5	.47
Time : Epistemic job demands (TAT)	0.010	4.2	.04
Time : Investigative education/occupation (RIASEC)	0.006	1.5	.21
Time : Epistemic leisure activities	0.023	5.6	.02
Time : Mindset	−0.013	9.7	.00
Time : Cognitive ability	−0.053	67.8	.00

**Table 3:** Results from mixed model analyses for predicting development of the variables depicted in the first column by Intellect. All results are from separate analyses.

Fixed Effect	Estimate	*F*	*p*
Epistemic behavior at work (PIAAC)	0.013	1.0	.32
Epistemic job demands (TAT)	0.003	0.0	.89
Investigative education/occupation (RIASEC)	0.021	1.9	.17
Epistemic leisure activities	0.030	3.7	.05
Mindset	0.018	0.8	.36
Cognitive ability	0.041	21.9	.00

## Data Availability

All data and scripts are available online via https://osf.io/jw3t9/ (accessed on 5 October 2024).
